# Are Landing Patterns in Jumping Athletes Associated with Patellar Tendinopathy? A Systematic Review with Evidence Gap Map and Meta-analysis

**DOI:** 10.1007/s40279-021-01550-6

**Published:** 2021-09-23

**Authors:** Abdulhamit Tayfur, Arman Haque, Jose Inacio Salles, Peter Malliaras, Hazel Screen, Dylan Morrissey

**Affiliations:** 1 Sports and Exercise Medicine, William Harvey Research Institute, QMUL, London, UK; 2 Barts Health NHS Trust, London, UK; 3 Department of Physiotherapy, Monash University, Melbourne, Australia; 4 School of Engineering Material Sciences, QMUL, London, UK

## Abstract

**Background:**

Patellar tendinopathy (PT) is common and debilitating for jumping athletes. Intriguingly, despite its high prevalence and many research studies, a causal explanation for PT presence remains elusive.

**Objective:**

Our objective was to investigate whether landing biomechanics among jumping athletes are associated with PT and can predict onset.

**Methods:**

We conducted a systematic review with evidence gap map and meta-analysis. We searched three databases from inception to May 2021 for observational studies or trials evaluating landing biomechanics in jumping athletes with PT (JPTs). We assessed quality with a modified Downs and Black checklist, risk of bias with the Quality Assessment of Diagnostic Accuracy Studies (QUADAS-2) tool, and evidence levels with van Tulder’s criteria and provided an evidence gap map.

**Results:**

One prospective cohort (moderate quality), one cross-sectional cohort (moderate quality), and 14 case–control (four high-, seven moderate-, and three low-quality) studies, including 104 JPTs, 14 with previous PT, 45 with asymptomatic patellar tendon abnormality (PTA), and 190 controls were retained. All studies had a high risk of bias. Meta-analysis showed an association between lower ankle dorsiflexion and the presence of tendinopathy during drop and spike landings, and JPTs had reduced knee joint power and work during volleyball approach or drop landings (moderate evidence). Limited evidence suggested that JPTs had lower patellar tendon loads during drop landings. Strong or moderate evidence showed no relation between PT and sagittal plane peak knee and hip angles or range of motion; hip, knee, or ankle angles at initial contact (IC); knee angular velocities, peak trunk kinematics, or trunk angles at IC; sagittal plane hip, knee, or ankle moments; and peak vertical ground reaction force (vGRF) and vGRF impulse. Identified gaps were that no study simultaneously investigated athletes with previous PT, current PT, and PTA, and studies of joint angular velocities at IC, ankle and hip angular velocities after touchdown, leg stiffness, loading rate of forces, and muscle activation are lacking.

**Conclusion:**

Despite the voluminous literature, large number of participants, multitude of investigated parameters, and consistent research focus on landing biomechanics, only a few associations can be identified, such as reduced ankle dorsiflexion–plantarflexion range. Further, the quality of the existing literature is inadequate to draw strong conclusions, with only four high-quality papers being found. We were unable to determine biomechanical factors that predicted PT onset, as longitudinal/prospective studies enabling causal inference are absent. The identified gaps indicate useful areas in which to explore causal relationships to inform intervention development. Therefore, high-quality prospective studies are essential to definitively determine whether landing biomechanics play a part in the development, recurrence, or management of PT and represent a potential therapeutic or preventive target alongside non-biomechanical factors.

**Supplementary Information:**

The online version contains supplementary material available at 10.1007/s40279-021-01550-6.

## Key Points

**Table:** 

Landing biomechanics may be associated with patellar tendinopathy (PT), but the level of evidence for the majority of variables was limited or very limited with a high risk of bias.
Only limited guidance can be given to reduce landing stiffness by using soft landing patterns integrated with improving the ankle dorsiflexion–plantarflexion range and optimising truncal–flexion strategies.
High-quality prospective studies are essential to gain strong evidence and identify causal relationships between jump-landing biomechanics and development or prognosis of PT.

## Introduction

Patellar tendinopathy (PT) is common, recurrent, and debilitating for competitive jumping athletes [1]. Across different sports, PT has an overall prevalence of approximately 14% of all injuries in elite athletes and 45 and 32% in volleyball and basketball, respectively [2]. For non-elite athletes, the prevalence ranges from 2.5 to 14.4% across various sports [3]. Recovery rates for PT are not satisfactory [4, 5], causing significant time off sport [5, 6] and recurrence rates of > 25% [6]. Up to 50% of athletes who are diagnosed with PT end their sports career because of long-term recalcitrant PT [4]. Intriguingly, despite the high prevalence and many research studies, causal explanations of PT non-recovery and recurrence remain elusive [7].

The knee has a major role in transferring load and dissipating mechanical energy during landing [8]. A high proportion of this load is transmitted through the patellar tendon [8] helping the lower limb joints to distribute kinetic energy [9], which has been proposed as one of the causal biomechanical factors for PT onset. Increased vertical jump performance (height) has previously been found to be a possible associated factor for PT in volleyball players, but evidence is only limited [10]. The mechanism is likely to be higher knee loads during higher jumps [10], which highlights the potential importance of landing patterns in jumping athletes. An association between altered landing kinematics and PT onset was previously reported [11]. Thus, landing biomechanics, including kinematics (e.g. initial contact angles of joints, peak joint angles, or angular velocities) and kinetics (e.g. joint moments, ground reaction forces, tendon forces, or lower limb muscle activation patterns), are plausible factors that may influence PT onset or become impaired following PT onset. Therefore, synthesising study results concerning landing biomechanics is necessary.

Van der Worp et al. [11] conducted a systematic review with six studies reporting horizontal landing kinematics potentially linked to PT onset. Harris et al. [12] published an updated systematic review of 15 studies finding 37 biomechanical variables to be associated with PT and asymptomatic patellar tendon abnormality (PTA) but undertook no grading of evidence level or pooling of data, limiting data interpretation. De Bleecker et al. [13] published a systematic review with meta-analysis investigating jump-landing kinematics for a range of lower extremity overuse injuries, including nine reports specific to PT, and concluded that the kinematic associations with PT are poorly understood. No recent comprehensive review has scoped the literature to demonstrate evidence gaps (as per established approaches [14, 15]), graded the evidence, assessed the risk of bias, and pooled data from a comprehensive search of the literature. An updated review that addresses these deficits would help make sense of the literature for professionals attempting to manage and prevent PT.

The primary aim of this review was to determine whether jump-landing biomechanics are altered among jumping athletes with PT (JPTs) and can predict onset. A secondary aim was to quantify research quality and identify gaps in the literature to synthesise evidence regarding the role of jump-landing biomechanics in PT and guide future research.

## Methods

The Preferred Reporting Items for Systematic Reviews and Meta-Analyses (PRISMA) statement guided the design and reporting of this systematic review [16].

### Search Strategy

PubMed, Web of Science, and the Cochrane Library databases were searched from inception to May 2021. We used two domains in the search strategy with the following terms: patellar tendinopathy OR tendinitis OR tenosynovitis OR tendinosis OR other relevant synonyms for the condition domain AND jumping OR landing OR biomechanics for the task and measurements domain. Detailed search terms used can be found in Appendix S1 in the electronic supplementary material (ESM). No limits such as ‘time’ or ‘human studies’ were applied to the search.

### Inclusion and Exclusion Criteria

Interventional, cross-sectional, case–control, and prospective cohort studies in the English language investigating the association between three-dimensional landing biomechanics and PT were considered for inclusion. Case reports, case series, meetings, letters, editorials, reviews, pilot studies, abstracts, and animal studies were excluded. We included studies in jumping athletes (any sport) with a history of PT (or synonyms: tendinitis/tenosynovitis/tendinosis), and/or PT diagnosed clinically, and/or PTA assessed on ultrasound imaging, and/or healthy controls with or without assessment of tendon morphology. Studies of athletes with PTA were considered eligible for this review as this abnormality has been shown to be a risk factor for PT development [17, 18], hence potentially improving understanding of the associations with landing biomechanics. Measures of interest included kinematic variables such as initial contact angles of joints (hip, knee, ankle) or segments (i.e. trunk), range of motion (RoM) and peak angles in the same joints or segments, and joint angular velocities; and kinetic variables such as joint moments, peak ground reaction forces (GRF) in both horizontal and vertical planes, peak patellar tendon force (PTF), and lower limb muscle activation patterns.

### Study Selection

All studies identified by the search strategy were downloaded independently by two authors (AT and AH) into Mendeley Desktop (version 1.19.5, Mendeley Ltd., London, UK). After removing duplicates, two authors independently screened all titles and abstracts and retained the papers according to inclusion criteria. The full texts of papers retained during the screening of titles and abstracts alone were obtained and evaluated for final inclusion, and any disagreements were resolved at a consensus meeting with a third author (DM). Reference lists and citing articles of retained manuscripts were checked.

### Quality Assessment

The methodological quality of the included studies was assessed by two authors (AT and AH) using a 16-part adapted Downs and Black checklist (Table 1) with a maximum score of 17, with questions suited to intervention trials excluded, as has previously been utilised [19]. Scores of ≥ 13 (> 75%), 11–12 (60–74%), and ≤ 10 (< 60%) were taken to indicate high, moderate, and low quality, respectively [20, 21]. For prospective studies, items 9 and 26 were retained as they concern follow-up. Thus, we used an 18-part checklist with corresponding scores to assess prospective cohort studies only (Table 1). Additionally, for item 5, we considered age, sex, activity levels, height, and mass or body mass index as a confounding factor for scoring.

**Table 1:**
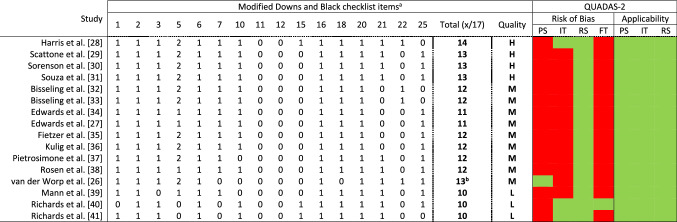
Results of the quality, risk-of-bias, and applicability assessments of the included studies

Red indicates high risk, green indicates low risk. For modified Downs and Black checklist items 1–3, 6, 7, 10–12, 15, 16, 18, 20, 21, 22, and 25: 0 = no or unable to determine, 1 = yes. For item 5: 0 = no, 1 = partially, 2 = yes

*FT* flow and timing, *H* high, *IT* index text, *L* low, *M* moderate, *PS* patient selection, *QUADAS-2* Quality Assessment of Diagnostic Accuracy Studies, *RS* reference standard

^a^Modified Downs and Black checklist items: 1 = clear aim, 2 = outcome measures described, 3 = participant characteristics described, 5 = confounding variables described, 6 = main findings described, 7 = measures of random variability provided, 10 = actual probability values reported, 11 = participants are representative of the population, 12 = confounders comparable between study groups and the source population, 15 = blinding assessors, 16 = analyses performed were planned, 18 = appropriate statistics, 20 = valid and reliable outcome measures, 21 = appropriate case–control matching (same population), 22 = participants recruited over the same time period, 25 = adjustment made for confounding factors. van der Worp et al. [26]: item 9 = 1; item 26 = 1, 13 of 19 in total, which is moderate

^b^Items used for prospective studies only: 9 = characteristics of patients lost to follow-up, 26 = numbers of patients lost to follow-up: 0 = none or unable to determine, 1 = yes

### Risk-of-Bias Assessment

Two authors (AT and AH) assessed the risk of bias for each included study using the Quality Assessment of Diagnostic Accuracy Studies (QUADAS-2) [22] tool. QUADAS-2 is strongly recommended for risk-of-bias assessment [23], utilizing diagnostic accuracy study criteria [22]. This approach was taken because the main aim of the included studies was to distinguish people with and without the condition. QUADAS-2 consists of four key domains covering patient selection, flow and timing, index test, and reference standard (3D biomechanical tests and clinical diagnosis in this instance). Domains are assessed in terms of risk of bias and applicability, yielding two judgements. These are stringently judged, with bias judged as high or unclear on at least one domain being designated at risk of bias or as having concerns regarding applicability [22].

### Data Extraction

Descriptive information was extracted from all included studies independently by two authors (AT and AH). This included publication details (author, year, study design), sample sizes, participant characteristics, the jumping task, and biomechanical outcomes (i.e. kinematics, kinetics, and muscle activation patterns) (Table 2). The biomechanical data for each outcome required to calculate effect sizes (mean and standard deviation) were extracted and corresponding authors contacted for additional data when needed. We used WebPlotDigitizer (https://automeris.io/WebPlotDigitizer/) to extract data when they were only presented in graphs.

**Table 2:** Study characteristics

Study	Population (n)	Demographics	Tasks implemented	Kinetic and kinematic measurements
Harris et al. [28], case–control	19 junior basketball players (9 M, 10 F), PT (8), C (11)	PT: 16.5 ± 0.6 y; 191.4 ± 14.4 cm; 78.7 ± 15.1 kg, 1.4 ± 1.1 h/w C: 15.9 ± 0.7 y; 183.7 ± 10.9 cm; 73.9 ± 9.7 kg, 2.4 ± 1.0 h/w Sporting level: elite	Stop-jump horizontal landing phase	Peak PTF and GRFs/LR of PTF and vGRF/peak force timing/peak net internal joint moments/impulses/peak joint angles and RoM (ankle, knee, hip, L5-S1 [lumbopelvis], and T12–L1 [thoracolumbar])
Scattone et al. [29], case–control	21 M volleyball and basketball players, PT (7), PTA (7), C (7)	PT: 18.0 ± 1.2 y; 189 ± 5 cm; 80.2 ± 7.9 kg, 14.4 ± 7.3 h/w PTA: 21.0 ± 5.2 y; 194 ± 11 cm; 90.8 ± 13.7 kg, 11.0 ± 6.5 h/w C: 16.3 ± 1.4 y; 196 ± 10 cm, 82.3 ± 10.9 kg, 15.3 ± 6.9 h/w Sporting level: elite	Bipedal drop landings from a 50-cm bench in three different trunk positions: self-selected, extended, and flexed	Ankle DF, knee, hip, and trunk flexion angles (at IC and peak angles)/peak vGRF and PTF/peak ankle PF and peak knee and hip extensor moments/forward head projection/knee pain
Sorenson et al. [30], case–control	13 M volleyball players, PT (6), C (7)	PT: 29.3 ± 4.1 y; 196 ± 5 cm; 92.8 ± 4.0 kg C: 24.3 ± 8.0 y; 197 ± 11 cm; 91.8 ± 5.8 kg Sporting level: elite	Maximum-effort volleyball approach jumps	Peak, average, and time-integrated vGRF/knee RoM, net joint work, and average net joint power/peak and average net joint moment/joint angular velocity
Souza et al. [31], case–control	14 M volleyball players, PT (7), C (7)	PT: 28.9 ± 4.5 y; 197 ± 11 cm; 91.7 ± 5.9 kg C: 24.9 ± 7.8 y; 197 ± 14 cm; 94.4 ± 6.4 kg Sporting level: elite	20 successive continuous hops on the dominant side. Only stance phase of task was used for analysis	Sagittal plane net joint moments (ankle, knee, hip)/total support moment (sum of the averaged hip and knee extensor and ankle plantar flexor net joint moments)/joint contributions to the total support moment
Bisseling et al. [32], case–control	24 M volleyball players, previous PT (7), recent PT (9), C (8)	Previous: 22.4 ± 2.6 y; 189 ± 7 cm; 79.5 ± 5.6 kg Recent: 24.1 ± 3.3 y; 192 ± 6 cm; 85.0 ± 10.1 kg C: 23.6 ± 2.5 y; 189 ± 8 cm; 84.5 ± 13.2 kg All training at least three times weekly and had been competitive ≥ 5 y	Drop landings with various high platforms (30, 50, 70 cm)	Peak and loading rate vGRF/joint flexion angles, angular velocity, peak moments/loading rates of ankle and knee moments/joint power and work
Bisseling et al. [33], case–control	15 M volleyball players, previous PT (7), C (8)	Previous: 22.4 ± 2.6 y; 189 ± 7 cm; 79.5 ± 5.6 kg, 7.9 ± 4.0 h/w C: 23.6 ± 2.5 y; 189 ± 8 cm; 84.5 ± 13.2 kg, 7.7 ± 2.7 h/w Sporting level: elite and non-elite	Spike jump, dominant foot landing	Joint maximum angle, RoM touchdown till peak vGRF, angles at IC and peak vGRF/joint velocities/ankle and knee moments/LR of knee extensor moment and vGRF
Edwards et al. [34], case–control	14 M athletes from team sports, PTA (7), C (7)	PTA: 25.2 ± 4.7 y; 183.4 ± 7.2 cm; 83.2 ± 9.0 kg C: 22.3 ± 2.4 y; 185.9 ± 8.1 cm; 82.0 ± 12.6 kg Sporting/training level not reported	Five stop-jumps involving a simultaneous two-foot horizontal and vertical landing	Peak PTF and vGRF/LR of PTF and vGRF/joint kinematics/time of onset and peak muscle activity relative to peak PTF time
Edwards et al. [27], cross-sectional	Seven players with PTA, volleyball (1), basketball (4), soccer (2)	25.2 ± 4.7 y; 183.4 ± 7.2 cm; 83.2 ± 9.0 kg Sex and sporting/training level not reported	Five stop-jump task trials before and after a fatigue protocol	Peak vGRF and anterior–posterior GRF/3D lower limb kinematics/net peak PTF/net internal peak knee moment/LR of PTF and vGRF
Fietzer et al. [35], case–control	18 dancers (9 M, 9 F), PT (6), C (12)	PT: 18.8 ± 0.8 y; 172 ± 11 cm; 66.9 ± 7.3 kg C: 18.9 ± 1.2 y; 168 ± 8 cm, 59.2 ± 9.1 kg Pre-professional training programme (elite)	Eight saut de chat landings	GRF/joint landing angles and velocity
Kulig et al. [36], case–control	18 M volleyball players, PT (9), C (9)	PT: 25.9 ± 6.2 y; 195 ± 5 cm; 89.7 ± 6.6 kg C: 23.1 ± 7.3 y; 197 ± 10 cm; 94.1 ± 7.3 kg Sporting level: elite	Three successful spike-jump landings	GRFs and impulses/sagittal plane joint angles (ankle, knee, hip) at IC and during the maximal knee flexion/the lower extremity contact angle (novel)
Pietrosimone et al. [37], case–control	41 M young athletes, PT (13), PTA (14), C (14)	PT: 19.6 ± 1.6 y; 182 ± 5 cm; 83.5 ± 5.1 kg; 8.0 ± 1.0 Tegner scale PTA: 21.0 ± 2.0 y; 184 ± 7 cm; 81.6 ± 13.0 kg; 8.0 ± 1.0 Tegner scale C: 19.6 + 1.6 y; 184 ± 9 cm; 79.9 ± 13.0 kg; 8.0 ± 0.9 Tegner scale	Five trials of a double-leg jump-landing task from a 30 cm box	PTF and GRF/knee and hip joint moments/PTF impulse/internal knee extension moment impulse/knee power and work
Rosen et al. [38], case–control	60 volleyball players, PT (30, 15 M, 15 F), C (30: 15 M, 15 F)	PT: 21.3 ± 3.2 y; 174.5 ± 9.4 cm; 72.8 ± 12.4 kg C: 21.5 ± 3.0 y; 174.9 ± 10.5 cm; 72.0 ± 14.7 kg All recreational ≥ 90 min of physical activity per week at ≥ 4 on Tegner scale	Five trials of a 40-cm, two-legged drop landing, followed immediately by a 50% maximum vertical jump	Joint angles at IC/peak joint angles/maximum angular displacement
Van Der Worp et al. [26], prospective cohort	49 basketball, volleyball, korfball players, PT (3: 2 M, 1 F), C (46: 30 M, 16 F)	M: 21.8 ± 3.5 y; 196 ± 7 cm; 86.2 ± 10.4 kg F: 21.6 ± 2.7 y; 178 ± 7 cm; 68.3 ± 10.7 kg All teams played at third or higher highest national level (elite and sub-elite)	A jump-landing-rebound task from a 30-cm high box at the start of each season (follow-up for two seasons [*n* = 18] and 1 season [*n* = 31])	At baseline and at end: joint angles/angle between foot and ground for IC phase between landing from horizontal jump and take-off of the vertical jump/leg stiffness
Mann et al. [39], case–control	20 M junior basketball players, PTA (10), C (10)	For 22 athletes: 17.7 + 1.5 y; 183 + 10 cm; 78.0 + 14.7 kg. Unknown for groups, but reported matched Sporting level: pre-elite	Five successful stop jumps	Sagittal plane knee and hip joints and trunk segment kinematics at IC and at the maximal knee flexion, plus hip flexion RoM
Richards et al. [40], case–control	10 M volleyball players, PT (3), C (7)	23.2 ± 0.8 y; 197.6 ± 1.9 cm; 91.9 ± 1.2 kg Sporting level: elite	Block jump-landing phases with one step approach. Spike jump-landing with only one foot hitting force plate	Maximal vGRF/knee moments and kinematics/knee (flexion, adduction, abduction) and tibial (IR and ER) angles
Richards et al. [41], case–control	10 M volleyball players, PT (3), C (7)	23.2 ± 0.8 y; 197.6 ± 1.9 cm; 91.9 ± 1.2 kg Sporting level: elite	A series of spike jump landings	Ankle DF, PF, inversion and eversion angles and moments/tibial IR and ER angles and moments

Values for age, height, mass, and training time are presented as mean ± standard deviation

*PTA* asymptomatic patellar tendon abnormality, *C* control, *DF* dorsiflexion, *ER* external rotation, *F* female, *GRF* ground reaction force, *h/w* training hours per week, *IC* initial contact, *IR* internal rotation, *LR* loading rate, *M* male, *PF* plantarflexion, *PT* patellar tendinopathy, *PTF* patellar tendon force, *RoM* range of motion, *vGRF* vertical GRF, *y* years

### Data Analysis

Quantitative analysis was conducted if the pooled data were methodologically homogeneous using random-effects models. Heterogeneity was further analysed with I^2^ and was considered as low (> 25–50%), moderate (> 50–75%), or high (> 75%) [24]. We used the Cochrane Review Manager software (version 5.3. Copenhagen: The Nordic Cochrane Centre, the Cochrane Collaboration, 2014) for the meta-analysis.

### Levels of Evidence

Based on the quality assessment, each variable of interest was assigned a level of evidence according to recommendations made by van Tulder et al. [25]:

1. Strong evidence: pooled results derived from three or more studies, including a minimum of two high-quality studies that were statistically homogenous (I^2^ not significant at 0.05); may be associated with a statistically significant or non-significant pooled result.
2. Moderate evidence: statistically significant pooled results derived from multiple studies that were statistically heterogeneous (*p* < 0.05), including at least one high-quality study, or from multiple moderate- or low-quality studies that were statistically homogenous (*p* > 0.05).
3. Limited evidence: results from one high-quality study or multiple moderate- or low-quality studies that are statistically heterogeneous (*p* < 0.05).
4. Very limited evidence: results from one moderate- or low-quality study.
5. Conflicting evidence: pooled results that are not significant and derived from multiple studies, regardless of quality, that are statistically heterogeneous (*p* < 0.05, i.e., inconsistent).

## Results

**Fig. 1:**
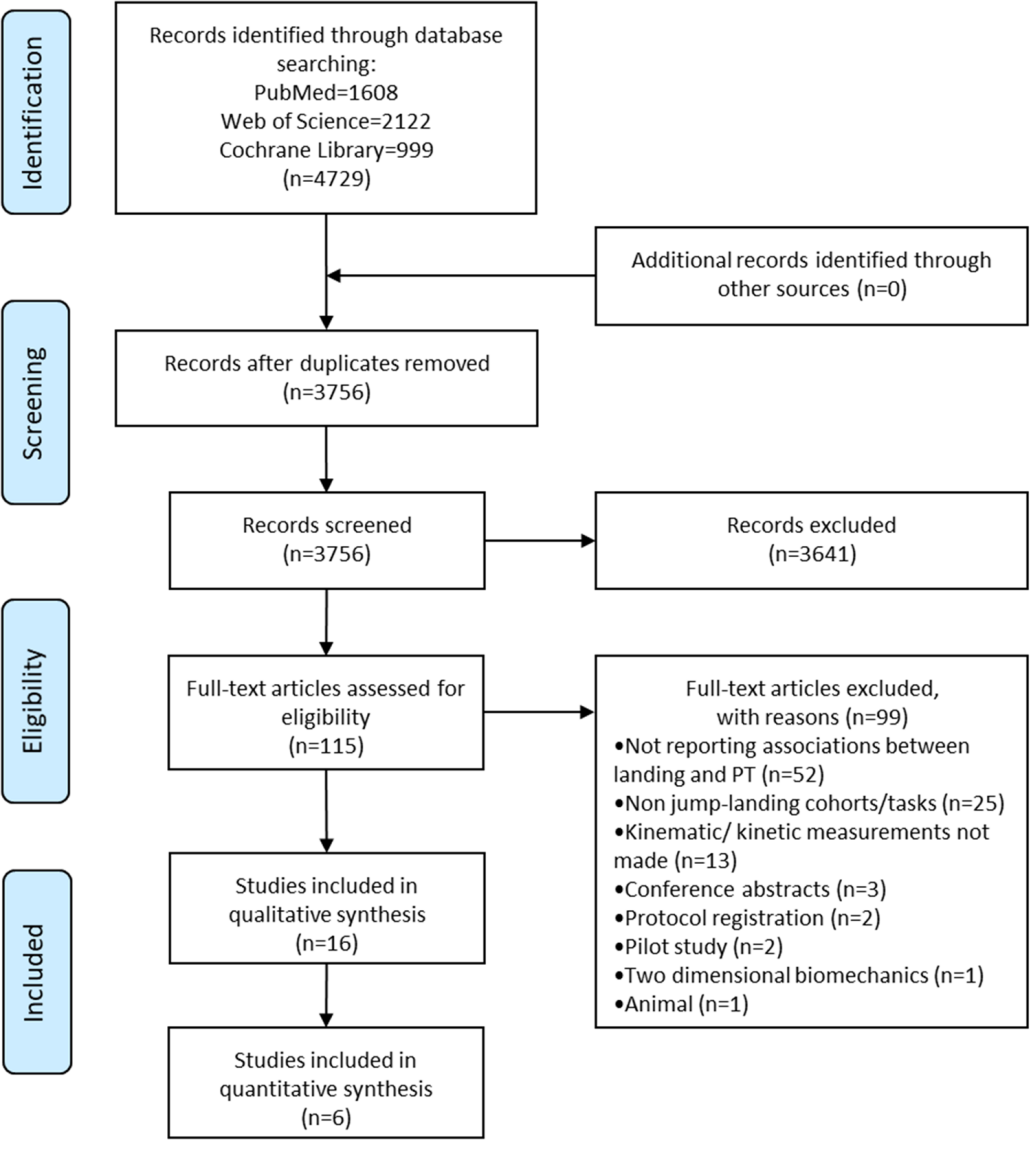
PRISMA flow diagram. *PT* patellar tendinopathy

Figure 1 shows the search results and study selection process. A total of 16 studies (one prospective cohort [26], one cross-sectional [27], and 14 case–control [28–41] studies) were included in the qualitative analysis. Studies included 104 JPTs, 14 with previous PT, 45 with PTA, and 190 controls. We were able to conduct only limited quantitative analysis because of methodological and outcome heterogeneity. After quality assessment, we identified four high-quality [28–31], nine moderate-quality [26, 27, 32–38], and three low-quality [39–41] studies. Quality assessment results and the characteristics of the included studies are shown in Tables 1 and 2, respectively. Risk-of-bias assessment and applicability results are contained in Table 1. All studies had a high risk of bias but low concerns regarding applicability. Specifically, only one study [26] had a low risk of bias for the ‘patient selection’ domain. All studies had a low risk of bias for the ‘reference standard’ domain, whereas only three studies [28, 40, 41] had a low risk of bias for the ‘index test’ domain. For the ‘flow and timing’ domain, only one study [40] had a low risk of bias, and the remainder had a high risk of bias.

### Levels of Evidence

**Fig. 2:**
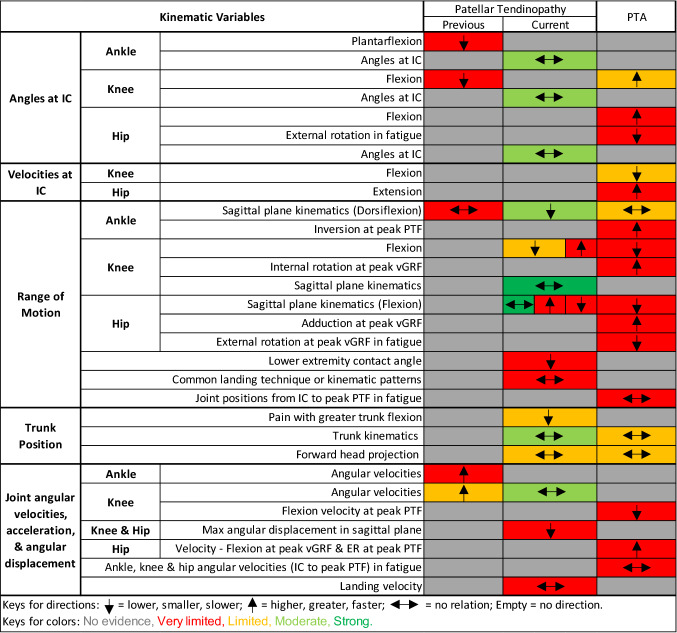
Evidence gap map for kinematics. Arrows show the direction of the variables associated with the condition. *ER* external rotation, *IC* initial contact, *max* maximum, *min* minimum, *PTA* asymptomatic patellar tendinopathy abnormality, *PTF* patellar tendon force, *vGRF* vertical ground reaction force

**Fig. 3:**
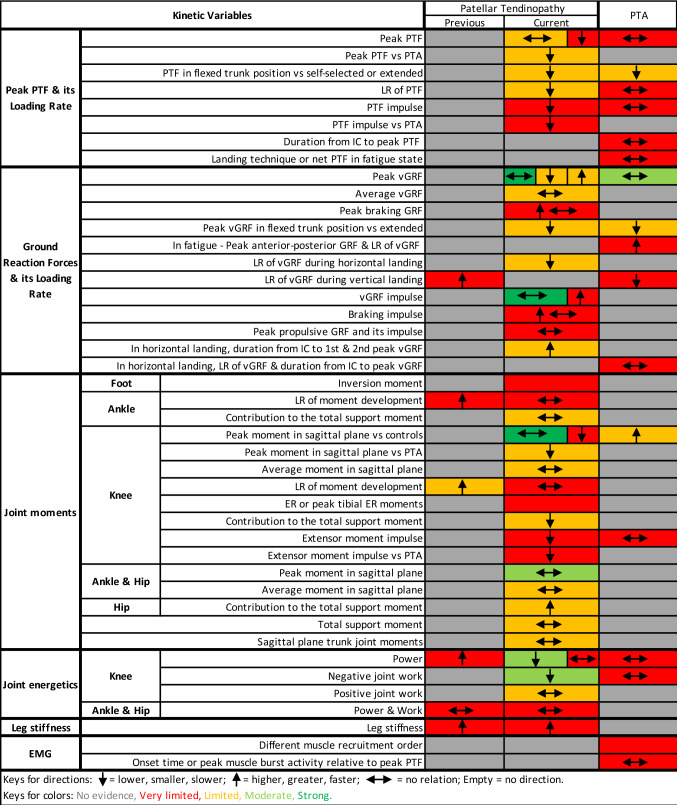
Evidence gap map for kinetics. Arrows show the direction of the variables associated with the condition. *EMG* electromyography, *ER* external rotation, *GRF* ground reaction force, *IC* initial contact, *LR* loading rate, *PTA* asymptomatic patellar tendinopathy abnormality, *PTF* patellar tendon force, *vGRF* vertical GRF

Findings and gaps in the literature are presented in Fig. 2 and Appendix S2 in the ESM for kinematics and in Fig. 3 and Appendix S3 in the ESM for kinetics with their relation to PT or PTA alongside their level of evidence. Here, we explore the findings of strong, moderate, or limited evidence in further detail than indicated in Figs. 2 and 3.

#### Kinematics

Strong evidence suggested no relation between PT and sagittal plane knee [28–30, 36] and hip [28, 29, 36] kinematics in peak joint angles or RoM, nor any relation between angle at peak vertical ground reaction force (vGRF) and PTF. Moderate evidence showed no relation between PT and hip, knee, or ankle joint angles at initial contact (IC) [28, 35, 36, 38], trunk kinematics (peak or angles at IC) [28, 29], and knee angular velocities [30, 32]. These variables were measured during drop landings [29, 32, 38], volleyball approach jump landings [30], spike jumps [36], stop-jump horizontal landings [28], and saut de chat landings [35].

**Fig. 4:**
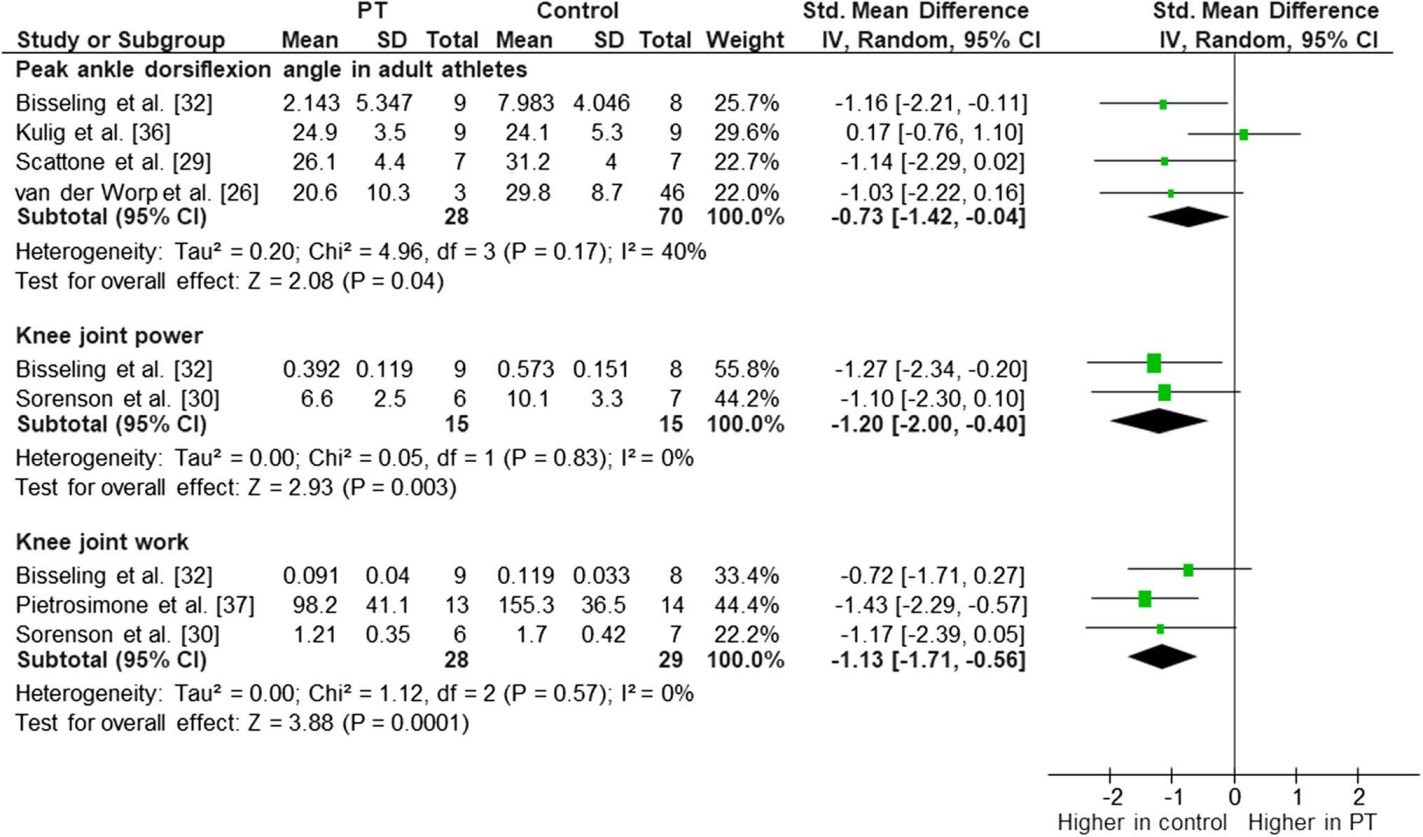
Meta-analysis for ankle dorsiflexion angle in adult athletes, knee joint power, and knee joint work (jumping athletes with current patellar tendinopathy symptoms vs. healthy controls). *CI* confidence interval, *df* degrees of freedom, *IV* inverse variance, *PT* patellar tendinopathy, *SD* standard deviation, *std* standard

We conducted a meta-analysis for ankle dorsiflexion RoM (Fig. 4) throughout the landing task, and moderate evidence indicated an association between lower peak dorsiflexion angle and PT (*I*^2^ = 40%, effect size − 0.73 [95% confidence interval {CI} − 1.42 to − 0.04]; *p* = 0.04) [26, 29, 32, 36] in adult athletes during multiple vertical jump-landing tasks consisting of spike and drop landings. When we pooled data for the drop landing task only, the association between smaller peak dorsiflexion angle with PT (*I*^2^ = 0%, effect size − 1.11 [95% CI − 1.76 to − 0.46]; *p* = 0.001) [26, 29, 32] was consistent. However, adding young athletes [28] (stop-jump horizontal landing phase) into the analysis increased the heterogeneity and eliminated the association with PT (*I*^2^ = 61%, effect size − 0.46 [95% CI − 1.21 to 0.28]; *p* = 0.22) [26, 28, 29, 32, 36]. Therefore, there was moderate evidence of smaller peak ankle dorsiflexion angle being associated with PT during multiple vertical jump-landing tasks in adult athletes (Fig. 4).

Limited evidence suggested a relation between higher knee angular velocity (mean difference range − 0.7 to − 0.6) [32, 33] and previous PT. There was a relation between PTA and greater knee flexion angle at IC (mean difference range 7.8°–14.1°) [34, 39], slower knee flexion velocities at IC (mean difference 183°/s) [34], and fatigue state (mean difference 70°/s) [27] (limited evidence). Limited evidence showed a relation between PT and lower knee flexion RoM (mean difference range 7.7°–8.6°) [26, 38] and greater hip flexion RoM (mean difference 11.3°) [26]. Additionally, limited evidence suggested a relation between trunk position and knee pain during drop landing in JPTs, as landing with greater trunk flexion decreased the pain immediately [29]. These variables were measured during drop landings [26, 29, 32, 38], spike jumps [33], and stop-jump horizontal or vertical landing phases [27, 34, 39].

Limited evidence showed no relation between PT and forward head projection [29] during drop landings or no relation between PTA and sagittal plane ankle kinematics [29] during drop landings.

#### Kinetics

Strong evidence indicated no relation between PT and peak vGRF [28, 29, 32, 36], vGRF impulse [28, 30, 36], and peak sagittal plane knee moments [28–30]. Moderate evidence showed no relation between PT and sagittal plane hip [28, 29] and ankle [28, 29] joint moments and no relation between PTA and peak vGRF [29, 34]. These variables were measured during drop landings [29, 32], volleyball approach jump landings [30], spike jumps [36], and stop-jump horizontal landings [28, 34].

In meta-analysis, moderate evidence indicated reduced knee joint power (*I*^2^ = 0%, effect size − 1.20 [95% CI − 2.00 to − 0.40]; *p* = 0.003) [30, 32] and work (*I*^2^ = 0%, effect size − 1.13 [95% CI − 1.71 to − 0.56]; *p* < 0.001) [30, 32, 37] in JPTs with current symptoms only versus healthy controls (Fig. 4) during volleyball approaches [30] or drop landings [32, 37].

Limited evidence indicated a relation between previous PT and higher loading rate of knee moment (mean difference range − 0.83 to 0.82) [32, 33]. Limited evidence also indicated associations between PT and lower loading rate of PTF (mean difference 16.3 BW·s^−1^) [28], lower loading rate of vGRF (mean difference 29.8 BW·s^−1^) [28], longer stance durations from IC to first and second peak vGRF (mean difference range 18.9–55.3) [28], smaller sagittal plane knee extensor moments compared with PTA (mean difference 0.07 N·m·N^−1^; *p* = 0.03; *d* = 1.77) [29], and greater hip (mean difference 8%) [31] and less knee (mean difference 8.4%) [31] contribution to the total support moment. Conflicting findings were detected as limited evidence showed both greater [35, 40] (36%; *p* < 0.001) [35] and lower [30] (22%; *p* = 0.003) [30] peak vGRF in JPTs. Furthermore, limited evidence indicated that PTF may have been related to both PT and trunk positions. There was a main effect of group (*p* = 0.048; *η*^2^ = 0.29) and of trunk position (*p* < 0.001; *η*^2^ = 0.56) in peak PTF [29]. Regardless of trunk position, JPTs had smaller peak PTF than athletes with PTA (*p* = 0.045; *d* = 0.98) [29], and landing with greater trunk flexion decreased the PTF immediately in symptomatic athletes [29]. Additionally, limited evidence suggested a relation between peak vGRF and trunk position as smaller peak vGRF was reported in landing with a flexed trunk position than extended (*p* = 0.043; *d* = 0.44) [29]. These variables were measured during drop landings [29, 32], volleyball approach jump landings [30], spike [33, 40] or block jumps [40], stop-jump horizontal landings [28], hopping [31], and saut de chat landings [35].

Limited evidence showed no relation between PT and peak PTF [28]; average vGRF [30]; average net ankle, knee and hip joint moments [31]; total support moment [31] or individual contributions of the ankle to the total support moment [31]; and sagittal plane lumbo-pelvic and thoraco-lumbar peak internal joint moments [28]. These variables were measured during volleyball approach jump landings [30], hopping [31], and stop-jump horizontal landings [28].

One of the key findings with very limited evidence was that athletes with previous [32] or current PT [26] presented with increased landing stiffness measured during drop landings.

## Discussion

We conducted this systematic review to determine whether jump-landing biomechanics were altered among JPTs and could predict onset to provide clinically applicable evidence-based information for professionals seeking to prevent and manage PT in jumping athletes. Our results indicated five strong (all non-associated), ten moderate (three associated and suitable for meta-analysis, seven non-associated), and 93 tentative (58 associated, 35 non-associated) findings. It is notable how few robust relevant positive findings were found, partly because the quality of the existing literature is too low and heterogeneous in terms of both methodology and outcomes to make useful clinical conclusions. However, a strength of our review is that we highlighted evidence gaps in the available literature, with a notable lack of adequately powered prospective studies to enable assessment of multi-factorial models.

Moderate evidence indicated an association between smaller ankle dorsiflexion range and current PT during vertical jump landing [26, 29, 32, 36]. Reduced ankle dorsiflexion angle was also clinically identified as a risk factor for PT onset [42, 43]. Ankle dorsiflexion has been shown to be a key shock-absorbing feature during landing [44]. During impact and throughout landing from a jump, eccentric calf muscle contraction accounts for 37–50% of the total kinetic energy absorbed by the muscular system [9]. Thus, a limitation in the dorsiflexion range might reflect altered landing biomechanics that impact on the presence of PT. Harris et al. [28] reported contradictory findings for ankle dorsiflexion range in junior athletes who performed stop-jump horizontal landings. Previous literature [11, 34, 39] showed that athletes presented with different landing strategies during horizontal and vertical landing tasks. This suggests that investigating the landing biomechanics in both horizontal and vertical phases of different sport-specific tasks is warranted. It is also plausible that landing patterns might differ in young athletes and adults, as young athletes are still growing and learning or improving the necessary techniques required for their sports. Therefore, we should also account for age alongside different types of tasks. However, it seems that findings from adults may not be readily applied to skeletally immature young athletes.

If knee flexion angles are greater at initial contact, the available RoM (further flexion) during landing is restricted, which will lead to decreased displacement of the centre of mass after initial contact and increased landing stiffness [11, 45]. It was suggested that increased landing stiffness may cause increased loading rates or forces on the patellar tendon [11]. Although findings with very limited evidence [26, 32] from this systematic review supported previous literature, indicating that athletes with previous or current PT presented with increased landing stiffness, limited evidence showed that JPTs presented with lower patellar tendon loading rates [28]. We also detected no association between PT and knee sagittal plane RoM [28–30, 36] (strong evidence) and knee angles at initial contact [28, 35, 36, 38] (moderate evidence). Overall, landing stiffness might be a factor contributing to PT, but the current evidence contradicts the potential explanations of observed stiffness. Therefore, future research investigating landing stiffness is needed to elucidate the association with PT.

JPTs had lower knee forces, resulting in reduced knee joint power [30, 32, 37] and work [30, 32] (moderate evidence), sagittal plane knee moments [29, 32], and patellar tendon loads [29, 37] (limited or very limited evidence). JPTs may modify their landing patterns to avoid higher patellar tendon loads and reduce their pain by minimising those knee forces. This should not be taken as a causal relationship, as it may represent reverse causality. A posited explanation is that athletes may load their contralateral side to protect the injured side and to avoid higher forces, as lower limb movement asymmetry has been shown during landing in male athletes with healthy patellar tendons [46]. On the other hand, training athletes using softer landing patterns could be one of the modifications. Studies have investigated leg stiffness by comparing a hard landing (stiff), a soft landing, and a normal landing [9, 47]. Although the stiffness measures were indirect, small decreases were found in knee moments but larger increases in knee angles during soft landings compared with hard landings [9, 47]. This indicates softer landings led to lower knee joint stiffness and reduced forces [45]. However, we found no association between PT and sagittal plane knee moments [28–30] (strong evidence) and patellar tendon loads [28] (limited evidence).

Trunk position may be related to PT indirectly as limited evidence indicated that JPTs used landing techniques with greater truncal flexion, which decreased pain and tendon forces during drop landings [29], although trunk flexion did not differ from that in controls [29]. Furthermore, greater truncal flexion increased peak knee and hip flexion angles during drop landings, despite decreased peak ankle dorsiflexion angles [29]. Relative to self-selected or extended trunk positions, a flexed trunk position also resulted in less vGRF and PTFs [29]. Therefore, a flexed trunk position may help decrease stiffness in knee and hip joints, and therefore might be a strategy for a soft landing pattern.

### Limitations

The quality of the existing literature exploring associations between jump-landing activities and PT was problematic, as 75% of the included papers were of moderate or low quality, and the risk of bias was high for all papers. Thus, the data did not provide strong evidence for the biomechanical factors of interest, and causal relationships remained unclear. The variability of the existing literature was also high in terms of differences in the tasks implemented, populations, and variables of interest measured. This high heterogeneity limited our ability to pool data for a meta-analysis of many parameters. Findings from this review were especially limited for female populations, as 11 of 16 studies only recruited males. We did not specify whether healthy control groups had to have undergone ultrasound assessment, so it is possible that some control participants in 11 of 16 studies might have had PTA. Although these would not alter the clinical diagnosis, it is plausible that they would have pre-clinical alterations in landing mechanics. However, we performed a sensitivity analysis for peak ankle dorsiflexion angle without any resultant change of findings. For completeness, we recommend future studies include ultrasound imaging. Additionally, in 13 of the 16 included studies, small sample sizes reduced the methodological quality as they did not provide the minimum requirement of ten ‘events per variable’ of interest [48].

### Future Directions

It is clear that definitive, adequately powered, well-designed prospective studies with high-quality measurements and adequate follow-up are required to determine whether jump-landing biomechanical factors play a part in the development, recurrence, and management of PT, alongside non-biomechanical factors. Additionally, high-quality prospective studies could also establish multi-factorial causality models to inform planned interventions, whereas randomized controlled trials (RCTs) could investigate the effects of movement strategies on risk reduction.

Many studies [28–30, 32, 35, 36, 38] identified factors that had a theoretically plausible relationship with PT but were not found to be associated. Strong or moderate evidence indicated that there was no relation between PT and sagittal plane peak knee and hip kinematics, lower limb joint angles at initial contact, trunk kinematics, knee angular velocities, peak vGRF and vGRF impulse, and peak sagittal plane lower limb joint moments. This systematic review showed that studies allowing causal inference were scarce, as most of the existing literature consisted of case–control studies, there being only one prospective cohort [26], and that with a problematically small sample size of JPTs.

Five studies included a PTA group (two comparing with PT and controls [29, 37], two comparing with controls [34, 39], and one including only PTA [27]), whereas only two studies included previous PT (one comparing with PT and controls [32] and one comparing with controls [33]). Based on the available evidence, these groups presented different biomechanical features compared with PT or controls in ankle and knee angles at IC, ankle dorsiflexion angle, knee angular velocity, and knee joint power and work, whereas they presented similar features in trunk kinematics, leg stiffness, and ankle and hip joint power and work. We also noted that no study simultaneously investigated participants with PTA, current PT, and previous PT, which could provide explanations for causal relationships, as this would take into consideration the time periods before, during, and after the condition. Nor have investigations of joint angular velocities at initial contact, ankle and hip angular velocities after touchdown, leg stiffness, loading rate of forces, and muscle activation in PT populations been performed. These would be useful areas in which to explore causal relationships with high-quality large prospective cohort studies.

The existing literature mainly focused on GRF, which represents total load on the lower limb. Of the 16 included studies, 11 investigated GRF (nine studies of PT [28–30, 32, 33, 35–37, 40] and four of PTA [27, 29, 34, 37]), whereas the number was lower for studies exploring knee moment (five studies of PT [29, 30, 32, 33, 40] and two of PTA [27, 29]) and PTF (three studies of PT [28, 29, 37] and four of PTA [27, 29, 34, 37]). We suggest that GRF might not be the ideal variable for JPTs because of the limitations of inverse dynamic modelling. There is a particular lack of study on tendon forces, which could provide improved understanding about force distribution and its relationship with PT. Therefore, we still need to know more about forces acting on the knee, especially PTF, as we know a high load is transmitted across the knee and that it is a primary shock absorber [8].

Future work should also consider non-biomechanical factors alongside biomechanical variables to identify covariates and interactions. Several intrinsic and extrinsic non-biomechanical risk factors increasing the onset of PT in athletes have been identified, with age, height, weight, sex, genetics, alignment of lower limb, flexibility, and increased jump height the main intrinsic factors reported [3, 26]. Extrinsic factors included practising a jumping sport characterized by high demands on speed and power for the leg extensors, the number of training hours (elite athletes > non-elite), amount of training, playing surface, number of jumps performed, playing position, and the high frequency and intensity of training and competition [3, 26, 49–51]. It seems plausible that the higher the mechanical overload on the tendon, the greater the risk for developing a PT [3], irrespective of the landing biomechanics. Therefore, future high-quality prospective studies simultaneously measuring plausible biomechanical and non-biomechanical variables—such as workload, clinical examination findings, and psychosocial factors—would be the key approach required to determine what part jump-landing biomechanical factors play in the development or treatment of PT.

### Clinical Implications

At present, only limited guidance can be provided for clinicians. Evidence is only moderate or limited; however, from this, we identified biomechanical variables that are clinically modifiable, to inform professionals managing and trying to prevent PT. Clinicians could initially focus on increasing ankle sagittal plane RoM to improve the absorption of the reaction forces from landing, potentially decreasing the load on the patellar tendon. Another approach would be increasing truncal flexion during landing as this may help reduce pain and tendon forces [29]. Lastly, working on soft landing patterns may be beneficial as this helps decrease landing stiffness by reducing knee joint moments and increasing knee RoM [9, 47]. The risks of such strategies in terms of performance reduction need to be considered, so an alternative approach would be to enhance the athlete’s capacity to deal with such forces during a session and maximise recovery strategies.

## Conclusion

Landing biomechanics may be associated with PT, but the level of evidence for the majority of variables was limited or very limited and the risk of bias high, despite good applicability. At present, only limited guidance for clinicians and coaches is warranted, with three recommendations that can be summarised around making landings less stiff, at least initially. Specifically, these are improving ankle dorsiflexion–plantarflexion range, optimising truncal–flexion strategies, and using soft landing patterns. The literature quality is currently insufficient for robust recommendations, with high-quality prospective studies now essential to determine whether jump-landing biomechanics play a part in the development or treatment of PT, alongside non-biomechanical factors. Further prospective studies could also establish multi-factorial causality, whereas RCTs could investigate the effects of movement strategies on risk reduction and recovery.

## Supplementary Information

Below is the link to the electronic supplementary material.

Supplementary file1 (DOCX 95 kb)

## Declarations

### Availability of data and material

The data that support the findings of this study are available on request from the corresponding author.
