# Supplementary material for: Are Landing Patterns in Jumping Athletes Associated with Patellar Tendinopathy? A Systematic Review with Evidence Gap Map and Meta-analysis
Source: Sports Med. 2021 Sep 23;52(1):123–37. doi: 10.1007/s40279-021-01550-6 (PMC8761156; doi:10.1007/s40279-021-01550-6)
Supplement: Supplementary file 1 — Supplementary file1 (DOCX 95 kb) [file 40279_2021_1550_MOESM1_ESM.docx]

**SUPPLEMENT**

**Supplementary Information Appendix S1: Search strategy for all databases**

**Latest search performed on 24.05.2020.**

**Pubmed:**

(“Tendinopathy” OR “tendinopathies” OR “tendinitis” OR “tendonitis” OR “tendinosis” OR “tenosynovitis” OR “paratendinopathy” OR “Paratenonitis” OR “noninsertional” OR “insertional” OR “Patella-tendon” OR “Patellar-tendon” OR “Patella-tendinopathy” OR "Patellar-tendinopathy" OR “Patella-tendinitis” OR “Patellar-tendinitis” OR “Patella-tendinosis” OR “Patellar-tendinosis” OR “Patella-paratenonitis” OR “Patellar-paratenonitis” OR “patella-apicitis” OR “patellar-apicitis” OR “patella-apex-syndrome” OR “patellar-apex-syndrome” OR “patella-tip-syndrome” “patellar-tip-syndrome” OR “patella-tenosynovitis” OR “patellar-tenosynovitis” OR “jumpers-knee” OR “Jumper’s-knee” OR “tendon injury”)

AND

(“jump” OR “jumping” OR “touchdown” OR “land” OR “landing” OR “kinematics” OR “biomechanics” OR “Mechanical-stress” OR “Mechanical-stresses” OR “take-off” OR “Jump-landing”)

**Web of Science Search:**

ALL FIELDS: (“Tendinopathy” OR “tendinopathies” OR “tendinitis” OR “tendonitis” OR “tendinosis” OR “tenosynovitis” OR “paratendinopathy” OR “Paratenonitis” OR “noninsertional” OR “insertional” OR “Patella-tendon” OR “Patellar-tendon” OR “Patella-tendinopathy” OR "Patellar-tendinopathy" OR “Patella-tendinitis” OR “Patellar-tendinitis” OR “Patella-tendinosis” OR “Patellar-tendinosis” OR “Patella-paratenonitis” OR “Patellar-paratenonitis” OR “patella-apicitis” OR “patellar-apicitis” OR “patella-apex-syndrome” OR “patellar-apex-syndrome” OR “patella-tip-syndrome” OR “patellar-tip-syndrome” OR “patella-tenosynovitis” OR “patellar-tenosynovitis” OR “jumpers-knee” OR “Jumper’s-knee” OR “tendon injury”)

AND

ALL FIELDS: (“jump” OR “jumping” OR “touchdown” OR “land” OR “landing” OR “kinematics” OR “biomechanics” OR “Mechanical-stress” OR “Mechanical-stresses” OR “take-off” OR “Jump-landing”)

**Cochrane Library:**

“Tendinopathy” OR “tendinopathies” OR “tendinitis” OR “tendonitis” OR “tendinosis” OR “tenosynovitis” OR “paratendinopathy” OR “Paratenonitis” OR “noninsertional” OR “insertional” OR “Patella-tendon” OR “Patellar-tendon” OR “Patella-tendinopathy” OR "Patellar-tendinopathy" OR “Patella-tendinitis” OR “Patellar-tendinitis” OR “Patella-tendinosis” OR “Patellar-tendinosis” OR “Patella-paratenonitis” OR “Patellar-paratenonitis” OR “patella-apicitis” OR “patellar-apicitis” OR “patella-apex-syndrome” OR “patellar-apex-syndrome” OR “patella-tip-syndrome” “patellar-tip-syndrome” OR “patella-tenosynovitis” OR “patellar-tenosynovitis” OR “jumpers-knee” OR “Jumper’s-knee” OR “tendon injury” in All Text

AND

“jump” OR “jumping” OR “touchdown” OR “land” OR “landing” OR “kinematics” OR “biomechanics” OR “Mechanical-stress” OR “Mechanical-stresses” OR “take-off” OR “Jump-landing” in All Text - (Word variations have been searched)

**No Additional Filters.**

**No language restrictions.**

**Sorted by ‘Most Recent’ in Pubmed.**

**All fields search in all databases.**

**RESULTS**

PUBMED: 1608

WOS: 2122

COCHRANE: 999

OVERALL: 4729

**Supplementary Information Appendix S2: Jump-landing kinematic analyses. Keys: H, high; M, moderate; L, low; PT, patellar tendinopathy; PTA, asymptomatic patellar tendon abnormality; JPTs, jumping athletes with PT; vGRF, vertical ground reaction force; LR, loading rate; DF, dorsiflexion; PF, plantarflexion; IR, internal rotation; ER, external rotation; RoM, range of motion; IC, initial contact; PTF, patellar tendon force.**

| **Kinematic variables** | **Findings** | **Quality (n)** | | | **Evidence Level** |
| --- | --- | --- | --- | --- | --- |
|  |  | **H** | **M** | **L** |  |
| **Joints angles at IC (ankle, knee, hip)** | Lower ankle PF, higher likelihood of previous PT [33]. |  | 1 |  | Very Limited |
|  | More limited knee flexion, higher likelihood of previous PT [33]. |  | 1 |  | Very Limited |
|  | PTA group landed with greater knee flexion [34,39]. |  | 1 | 1 | Limited |
|  | Hip angles may be related to PTA; a trend for greater hip flexion [34] & significantly smaller hip ER in fatigue [27]. |  | 2 |  | Very Limited |
|  | No relation between joint angles at IC and PT [28,35,36,38]. | 1 | 3 |  | Moderate |
| **Joints velocities at IC (knee, hip)** | Slower knee flexion velocity possibly related to PTA [27,34]. |  | 2 |  | Limited |
|  | Hip joint velocity may be related to PTA; hip extension displaying significantly faster hip extension velocity [34]. |  | 1 |  | Very Limited |
| **Joints RoM (ankle, knee, hip)** | An association of smaller ankle dorsiflexion with PT [26,29,32,36]. | 1 | 3 |  | Moderate |
|  | No relation between sagittal plane ankle kinematics and PT in young athletes [28]. | 1 |  |  | Limited |
|  | No relation between sagittal plane ankle kinematics and PTA [29]. | 1 |  |  | Limited |
|  | No relation between sagittal plane ankle kinematics and previous PT [32]. |  | 1 |  | Very Limited |
|  | PTA group had greater ankle inversion at PTF [34]. |  | 1 |  | Very Limited |
|  | JPTs had lower peak knee flexion angles [26,38]. |  | 2 |  | Limited |
|  | A deeper knee flexion predicted 100% cases for PT [40]. |  |  | 1 | Very Limited |
|  | PTA group had greater knee IR at vGRF and lower peak knee flexion angles [34]. |  | 1 |  | Very Limited |
|  | No relation between sagittal plane knee RoM and PT [28–30,36]. | 3 | 1 |  | Strong |
|  | JPTs had more hip flexion [26]. |  | 1 |  | Very Limited |
|  | JPTs had lower peak hip flexion [38]. |  | 1 |  | Very Limited |
|  | PTA group had lower hip flexion RoM [39]. |  |  | 1 | Very Limited |
|  | PTA group had greater hip adduction at vGRF [34]. |  | 1 |  | Very Limited |
|  | Athletes with PTA had smaller hip ER at vGRF when fatigued [27]. |  | 1 |  | Very Limited |
|  | No relation between PT and hip flexion RoM [28,29,36]. | 2 | 1 |  | Strong |
|  | JPTs had less Lower Extremity Contact Angle [36]. |  | 1 |  | Very Limited |
|  | JPTs (n=3) displayed no common landing technique or kinematic patterns [26]. |  | 1 |  | Very Limited |
|  | Similar joint positions from IC to peak PTF when fatigued in athletes with PTA [27]. |  | 1 |  | Very Limited |
| **Trunk Position** | Decreased pain with greater trunk flexion during landing in JPTs [29]. | 1 |  |  | Limited |
|  | No relation between PT and trunk kinematics [28,29]. | 2 |  |  | Moderate |
|  | No relation between PT and forward head projection [29]. | 1 |  |  | Limited |
| **Joint angular velocities, acceleration, & angular displacement** | Higher LR of ankle angular velocities in previous PT [33]. |  | 1 |  | Very Limited |
|  | A relation between higher knee angular velocity and previous PT [32,33]. |  | 2 |  | Limited |
|  | PTA group had a slower knee flexion velocity at PTF [34]. |  | 1 |  | Very Limited |
|  | JPTs had lower maximum knee angular displacement [38]. |  | 1 |  | Very Limited |
|  | Hip angular velocity [34] & angular displacement [38] might be related to PT. |  | 2 |  | Very Limited |
|  | No relation between knee angular velocity and PT [30,32]. | 1 | 1 |  | Moderate |
|  | Similar ankle, knee & hip angular velocities from IC to peak PTF in PTA when fatigued [27]. |  | 1 |  | Very Limited |
|  | No group difference in landing velocity [35]. |  | 1 |  | Very Limited |

**Supplementary Information Appendix S3: Jump-landing kinetic analyses. Keys: H, high; M, moderate; L, low; PT, patellar tendinopathy; PTA, asymptomatic patellar tendon abnormality; JPTs, jumping athletes with PT; vGRF, vertical ground reaction force; LR, loading rate; DF, dorsiflexion; PF, plantarflexion; IR, internal rotation; ER, external rotation; RoM, range of motion; IC, initial contact; PTF, patellar tendon force.**

| **Kinetic variables** | **Findings** | **Quality (n)** | | | **Evidence Level** |
| --- | --- | --- | --- | --- | --- |
|  |  | **H** | **M** | **L** |  |
| **Peak Patellar Tendon Force and its Loading rate** | JPTs had smaller PTF than PTA group [29]. | 1 |  |  | Limited |
|  | JPTs had smaller PTF than controls [37]. |  | 1 |  | Very Limited |
|  | No relation between PT and PTF [28]. | 1 |  |  | Limited |
|  | No relation between PTA and PTF [34,37]. |  | 2 |  | Very Limited |
|  | An association of greater truncal-flexion with decreased PTF [29]. | 1 |  |  | Limited |
|  | JPTs had lower LR of PTF [28]. | 1 |  |  | Limited |
|  | JPTs had less PTF impulse vs PTA and controls [37]. |  | 1 |  | Very Limited |
|  | PTA had similar PTF impulse vs controls [37]. |  | 1 |  | Very Limited |
|  | Similar LR of PTF and duration from IC to PTF between groups [34]. |  | 1 |  | Very Limited |
|  | No relation between fatigue state and landing technique or net PTF [27]. |  | 1 |  | Very Limited |
| **Ground Reaction Forces (GRF) and its Loading rate** | Greater peak vGRF might be related to PT [35,40]. |  | 1 | 1 | Limited |
|  | JPTs showed 22% lower peak vGRF [30]. | 1 |  |  | Limited |
|  | Greater peak braking GRF might be related to PT [35]. |  | 1 |  | Very Limited |
|  | No group difference in average vGRF [30]. | 1 |  |  | Limited |
|  | No relation between peak vGRF and PT [28,29,32,36]. | 2 | 2 |  | Strong |
|  | No relation between peak vGRF and PTA [29,34]. | 1 | 1 |  | Moderate |
|  | No relation between peak braking GRF and PT [36]. |  | 1 |  | Very Limited |
|  | Smaller vGRF in landing with a flexed trunk position [29]. | 1 |  |  | Limited |
|  | Athletes with PTA had a greater peak anterior-posterior GRF when fatigued [27]. |  | 1 |  | Very Limited |
|  | JPTs had lower LR of vGRF during stop jump horizontal landing [28]. | 1 |  |  | Limited |
|  | Higher LR of vGRF in athletes with previous PT [32] and with PTA when fatigued [27]. |  | 2 |  | Very Limited |
|  | PTA group had a lower LR of vGRF during vertical landing phase [34]. |  | 1 |  | Very Limited |
|  | Longer duration from IC to first and second peak vGRF between groups [28]. | 1 |  |  | Limited |
|  | Similar LR of vGRF & duration (IC to peak vGRF) during horizontal landing phase [34]. |  | 1 |  | Very Limited |
|  | Greater vertical (15%) & braking impulses (126%) in dancers with PT [35]. |  | 1 |  | Very Limited |
|  | No group difference in vGRF impulse [28,30,36]. | 2 | 1 |  | Strong |
|  | No group difference in peak propulsive GRF [35], propulsive [35] & braking impulse [36]. |  | 2 |  | Very Limited |
| **Joint moments** | Foot inversion moment might be related to PT [41]. |  |  | 1 | Very Limited |
|  | Higher LR of ankle moment development in previous PT [32]. |  | 1 |  | Very Limited |
|  | No relation between PT and ankle joint contribution to the total support moment [31]. | 1 |  |  | Limited |
|  | An association of smaller knee moments with PT vs healthy controls [32]. |  | 1 |  | Very Limited |
|  | An association of smaller knee moments with PT vs PTA [29]. | 1 |  |  | Limited |
|  | ER moment at the left knee might be implicated in PT [40]. |  |  | 1 | Very Limited |
|  | Peak tibial ER moment predicted 8/10 cases for PT [40]. |  |  | 1 | Very Limited |
|  | JPTs displayed lower knee joint contribution to the total support moment [31]. | 1 |  |  | Limited |
|  | A relation between higher LR of knee moment and previous PT [32,33]. |  | 2 |  | Limited |
|  | JPTs had less knee extensor moment impulse vs PTA & controls [37]. |  | 1 |  | Very Limited |
|  | PTA group had similar knee extensor moment impulse vs controls [37]. |  | 1 |  | Very Limited |
|  | JPTs displayed greater hip joint contribution to the total support moment [31]. | 1 |  |  | Limited |
|  | No relation between PT and peak knee moments [28–30]. | 3 |  |  | Strong |
|  | No relation between PT and peak ankle [28,29] and hip [28,29] moments. | 2 |  |  | Moderate |
|  | No relation between PT and average ankle, knee and hip moments [31]. | 1 |  |  | Limited |
|  | No relation between PT and total support moment [31]. | 1 |  |  | Limited |
|  | No relation between PT and sagittal plane trunk joint moments [28]. | 1 |  |  | Limited |
| **Joint energetics** | Lower knee joint power in JPTs [30,32]. | 1 | 1 |  | Moderate |
|  | Lower negative knee joint work in JPTs [30,32,37]. | 1 | 2 |  | Moderate |
|  | No group difference in positive knee joint work [30]. | 1 |  |  | Limited |
|  | JPTs & previous PT had similar and ankle & hip joints power & work [32]. |  | 1 |  | Very Limited |
|  | Similar knee joint power (PT & PTA) & work (PTA) [37]. |  | 1 |  | Very Limited |
| **Leg stiffness** | JPTs [26] and previous PT [32] had higher leg stiffness. |  | 2 |  | Very Limited |
| **Muscle activation** | Athletes with PTA might have different muscle recruitment order [34]. |  | 1 |  | Very Limited |
|  | No group differences in onset time or peak muscle burst activity relative to peak PTF [34]. |  | 1 |  | Very Limited |

**DECLARATIONS**

**Funding**

Postgraduate studies of Mr Abdulhamit Tayfur were sponsored by the Republic of Turkish Ministry of National Education. The sponsors had no role in study design, data collection and analysis, decision to publish, or preparation of the manuscript.

**Conflicts of interest/Competing interest**

Abdulhamit Tayfur, Arman Haque, Jose Inacio Salles, Peter Malliaras, Hazel Screen and Dylan Morrissey declare that they have no conflicts of interest relevant to the content of this review.

**Ethics approval**

Not applicable

**Consent to participate**

Not applicable

**Consent for publication**

Not applicable

**Availability of data and material**

The data that support the findings of this study are available on request from the corresponding author [DM].

**Code availability**

Not applicable

**Authors’ contributions**

AT, AH and DM conceived and designed the review. AT and AH screened the articles, extracted the data, provided methodological quality ratings and analysed the data. AT wrote the first draft of the manuscript. JAS, PM, HS and DM critically revised the manuscript. All authors read and approved the final manuscript for publication.
